# CD5: from antiquated T cell marker to immunotherapy’s new hope

**DOI:** 10.1038/s41392-023-01494-5

**Published:** 2023-05-25

**Authors:** Sandra Schwarz, Michael Linnebacher

**Affiliations:** Molecular Oncology and Immunotherapy, Clinic of General Surgery, Rostock University Medical Center, 18057 Rostock, Germany

**Keywords:** Tumour immunology, Preclinical research, Immunotherapy

In a recent study published in Science, He et al. investigated the role of CD5 on dendritic cells (DCs) in priming effector T cells.^1^ By proving the importance of CD5 in engaging effective antitumor immune responses, they highlighted the untapped potential of CD5 for targeted immunotherapy.

Checkpoint-inhibitor immunotherapy can safely be considered as a revolutionary development in cancer treatment. However, serious side-effects are frequent, prediction of therapy success is still challenging and nonresponding patients are omnipresent. Improved understanding of the interaction of antigen-presenting with effector cells, especially in the tumor microenvironment (TME), might lay the foundation for advanced therapeutic approaches. DCs, the most important antigen-presenting cells, are further divided in CD5^+^ and CD5^-^ subsets with differential capacity for T cell activation. Thus, He et al. scrutinized the functional role of CD5 in immunologic interactions and its prognostic value for patient survival.

**Fig. 1:**
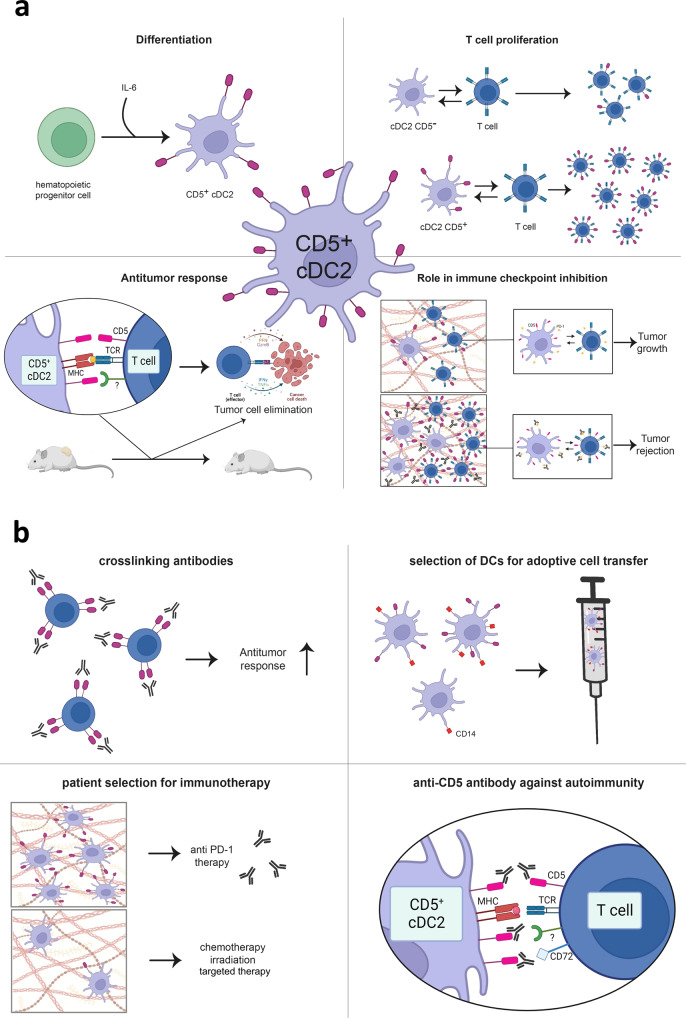
The role of CD5 in tumor immunology and therapeutic chances. **a** CD5^+^ cDC2 play an important role in tumor immunology. (upper left) Under influence of IL-6, hematopoietic progenitor cells differentiate into CD5^+^ cDC2. (upper right) CD5^+^ cDC2 induce strong proliferation of CD5^high^ T cells, while proliferation induced by CD5^-^ cDC2 is less effective and limited to CD5^low^ T cells. (lower left) Successful T-cell-induced tumor cell elimination depends on CD5 expression on both cDC2 and T cells. Beside interaction of CD5^+^ cDC2 with CD5 on T cells, yet unidentified receptors might be involved, too. (lower right) Anti-PD-1 therapy is most effective when CD5^+^ cDC2 are present in the TME and can induce activation and proliferation of CD5^+^ T cells. **b** CD5’s role in tumor immunology imply therapeutic chances. (upper left) The use of cross-linking antibodies could enhance antitumor response. (upper right) CD5 could be used for the selection of DCs instead of CD14 to improve T cell activating capacity. (lower left) Biopsy screening for CD5^+^ cDC2 might guide therapeutic decisions for immunotherapy. (lower right) In the autoimmune disease situation, blocking CD5 could regulate immune activity. cDC2 conventional dendritic cells 2, IL-6 interleukin 6, MHC major histocompatibility complex, PD-1 programmed cell death protein 1, TCR T cell receptor. Created with BioRender.com

One major finding was the induction of T cell proliferation by CD1c^+^ CD5^+^ DCs isolated from both skin and lymph nodes (see Fig. 1a). T cell proliferation was additionally accompanied by increased production of the activation markers IFNγ and TNFα as well as the effector molecules granzyme B and perforin. Moreover, T cell proliferation could further be improved by increased expression levels of CD5 on DCs. Beside the dependence of stimulating DC capacity on CD5, the authors also showed strengthened immune response against classical viral recall antigens (influenza, Epstein Barr and cytomegalovirus) when these were presented on CD5^+^ DCs. The stimulation of T cells with antigen-loaded CD5^-^ or CD14^+^ DCs was significantly less effective.

Further, in vivo experiments showed the functional dependence on CD5^+^ DCs for strong antitumor responses. When CD5^+^ DC were depleted, a majority of mice was unable to initiate a forceful immune reaction involving CD4^+^ and CD8^+^ effector T cells, while mice with unaltered DCs could and did successfully reject their tumors.

Aside from CD5’s role on DCs, the authors also elucidated CD5 expression on T cells and showed that it very closely mirrored the CD5 expression of DCs. It had been clear, that the antiquated T cell marker CD5 is important for T cell receptor signaling fine tuning and for protection from activation-induced cell death.^2^ Historically, CD5 has been considered as a purely negative regulator but more recent data imply, that via signaling through NF-κB, it helps maintaining higher levels of intracellular IκBα, which enables CD5^+^ T cells to better persist as effector/memory cells.^3^ Together with the data of the Klechevsky laboratory on CD5-mediated inhibition of apoptosis in DCs, this hints towards a general and assumedly context-dependent mechanism.

Remarkable differences regarding CD5-proficient and CD5-deficient immune cells were observed in experimental immunotherapy. The authors validated the necessity of CD5 on both DCs and T cells for noteworthy tumor regression induced by immune checkpoint inhibitor treatment (anti-PD-1 or anti-CTLA-4).

Further, He et al. validated older observations of reduced CD5 expression in the TME compared to healthy tissue, hinting at a tumor-induced downregulation.^4^ The parallel to specific modulation of known immune checkpoint molecules like PD-1 in the TME is striking. While immune checkpoints like PD-1 are expressed on T cells, they can also be found on CD5^+^ DCs. Taking the demonstrated utmost importance of CD5 into account, its induction by immune checkpoint inhibitors could restore tumor-suppressed DC activity via inducing CD5 expression and the proliferation of CD5^+^ DCs. The subsequent activation of tumor-reactive T cells could tip the scale back in the direction of equilibrium or even tumor elimination. This hypothesis is further strengthened by the increase of IL-6 in the TME of anti-PD1-treated mice and the observation of IL-6-dependent progenitor cell differentiation into CD5^+^ DCs.

The study of He and coworkers adds further important pieces to the puzzle of tumor-immune cell interaction in the TME of affected patients. Moreover, it raises a number of important questions like the role of CD5 on further immune cell types like B or NK cells and their subsets as well as their potential involvement in the CD5-dependent immune regulation. B cells with their strong antigen-presenting capacity are regularly present in the TME, but contrary to DCs and T cells, CD5^+^ B cells seem to be suppressive,^5^ thereby highlighting the duality in function of CD5, which must be clarified.

However, CD5^+^ DCs seem to be pivotal for immunotherapy success. Retrospective analyses of tumor patient cohorts treated with checkpoint inhibitors should be performed to clarify CD5’s therapeutic impact. If significantly correlating with response, CD5 could indeed be the desired biomarker to amend or even overcome the insufficient predictive significance of immune infiltration, PD-L1 expression or tumor mutational burden (see Fig. 1b).

For cellular therapy approaches, the results of He and coworkers have even stronger implications. Currently, DCs are mainly selected based on CD14 expression. If CD5 expression is better suited than CD14 to define T cell-activating DCs, CD5 determination or even selection should be taken into consideration for future DC vaccine preparation. As the authors also showed the positive effects of crosslinking and agonistic CD5 antibodies for boosting T cell responses, further research should focus on that, too. However, since CD5 might be a crucial factor for initiating strong immune reactions, this opens up the possibility of targeting this protein in overreacting immune cells present in autoimmunity (see Fig. 1b). Thus, CD5-targeted therapy approaches must be developed in a cell-specific and context-dependent manner to avoid undesirable interferences in the CD5^+^ cells’ multifunctional network.

With a positive prognostic value of the CD5^+^ DC signature observed for melanoma, lung squamous cell carcinoma, sarcoma, breast cancer, cervical squamous cell carcinoma and endocervical adenocarcinoma patient cohorts, CD5 could be of ubiquitous importance in a broad spectrum of tumors. But these data from The Cancer Genome Atlas (TCGA) need to be validated in independent clinical sample collections to round out this promising picture. It seems reasonable to assume that there will, for example, be clear differences between hypermutated and non-hypermutated subgroups of a given cancer type.

Ultimately, unraveling the presumable even more complex function of CD5 in cell fate decisions under pathological conditions, will clarify its duality in function and trigger therapeutic developments. Considering all aspects of CD5’s involvement in antitumoral immune response, the study of He and colleagues might well mark the emergence of a new phase particularly in immunotherapy.
